# Case Report: A rare case of giant gallbladder associated with adverse reactions to anlotinib

**DOI:** 10.3389/fmed.2025.1665373

**Published:** 2025-09-24

**Authors:** Jihao Cheng, Xin Wang, Jingyun Liao, Yuping Shen

**Affiliations:** ^1^Department of Radiology, The Second Affiliated Hospital of Zhejiang Chinese Medical University, Hangzhou, China; ^2^Department of Hepatobiliary Surgery, The Second Affiliated Hospital of Zhejiang Chinese Medical University, Hangzhou, China; ^3^Department of Radiology, Affiliated Xiaoshan Hospital, Hangzhou Normal University, Hangzhou, China; ^4^Department of Acupuncture and Moxibustion, The Second Affiliated Hospital of Zhejiang Chinese Medical University, Hangzhou, China

**Keywords:** giant gallbladder, chemotherapeutic drugs, anlotinib, case report, acute cholecystitis, adversereactions

## Abstract

Giant gallbladder (GGB) is a rare condition that is generally associated with biliary obstruction. GGB induced by chemotherapeutic agents have not been previously reported. This article presents a case of GGB potentially related to adverse effects of anlotinib. The patient was a 74-year-old female who had been taking anlotinib regularly for 2 years following the diagnosis of a malignant lung tumor. The patient was admitted because of abdominal pain accompanied by vomiting and discomfort for 11 days. No fever was observed during onset, but inflammatory markers were abnormally elevated, accompanied by impaired liver function. On physical examination, a tender mass was palpable in the right inguinal region. Abdominal computed tomography showed an enlarged gallbladder (18.6 × 7.2 × 5.3 cm). Enhanced magnetic resonance imaging and magnetic resonance cholangiopancreatography ruled out biliary stones or space-occupying lesions, leading to a final diagnosis of acute cholecystitis with GGB. The patient received antibiotic treatment and anlotinib was immediately discontinued. After 1 week of anti-infective therapy, the abdominal pain resolved, vomiting ceased, liver function and inflammatory markers returned to normal ranges, and the gallbladder regressed to a normal size. To our knowledge, this is the first reported case of GGB associated with anlotinib use. Through a literature review, we conducted an in-depth discussion on the pathogenesis of GGB and the pharmacological effects of anlotinib, with the aim of sharing experiences and alerting clinicians to such adverse events.

## Graphical abstract

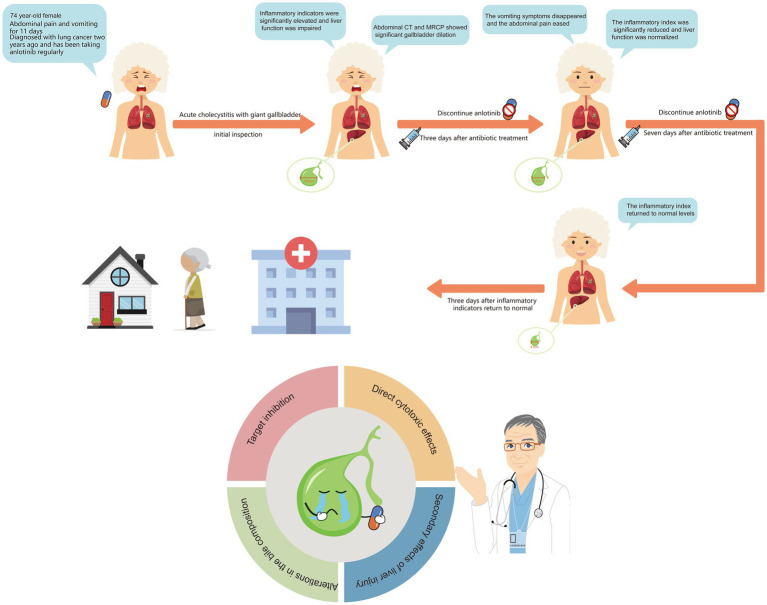

## Introduction

Cases of giant gallbladder (GGB) are exceedingly rare. A review of the literature spanning nearly 50 years yielded only 9 documented cases (Table 1). The pathogenesis underlying GGB formation remains unclear, although it is frequently caused by the obstruction of the biliary system by factors such as calculi, tumors, or parasites, which impede bile drainage and lead to near-unlimited gallbladder enlargement (1). To date, no case of anlotinib-induced GGB have been reported. However, occasional cases of gallbladder toxicity have been documented as adverse effects with other tyrosine kinase inhibitors (TKIs) such as sorafenib and sunitinib (2, 3).

**Table 1:** Case reports of giant gallbladders in PubMed over the past 50 years.

Case report	sex	Age (years)	Major comorbidity	GB size(cm)	GB volume	Underwent cholecystectomy
Maeda et al. (9)	36	NR	NR	NR	12.5 L	NR
Borodach et al. (26)	F	67	NR	20 × 12	1.5 L	Yes
Panaro et al. (14)	NR	17	Byler’s disease	43 × 21	2.7 L	Yes
Zong et al. (5)	F	55	NR	30 × 18	4.0 L	Yes
Kuznetsov et al. (4)	F	77	Chronic cholecystitis	24 × 17	3.3 L	Yes
Fultang et al. (27)	F	63	Chronic cholecystitis; Gallbladder stones	19 × 6	NR	Yes
Mirali et al. (28)	F	53	Chronic cholecystitis; Gallbladder stones	22 × 14	NR	Yes
Uemura al. (12)	M	82	Type 2 diabetes	18 × 7	NR	Yes
Gao et al. (1)	F	46	Chronic cholecystitis; Gallbladder stones	20 × 7	NR	Yes
This case	F	74	Chronic cholecystitis	19 × 7	NR	No

GB, gallbladder; F, female; M, male; NR, not record.

### Case description

**Figure 1:**
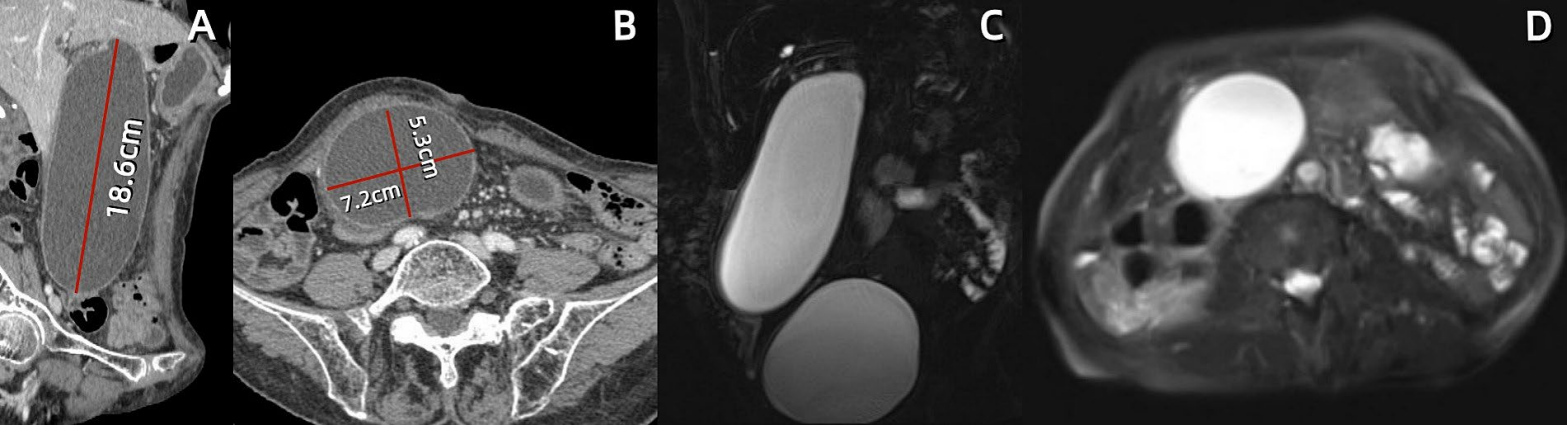
Abdominal contrast-enhanced computed tomography (CT) and magnetic resonance cholangiopancreatography (MRCP) at first hospitalization. **(A,B)** Sagittal and axial CT scans reveal a markedly enlarged gallbladder measuring approximately 18.7 × 7.2 × 6.1 cm, with the fundus extending deep into the right iliac fossa. **(C,D)** Coronal MRCP and axial T2-weighted images demonstrate a massive gallbladder mass beneath the distended urinary bladder.

A 74-year-old female presented to our hospital with abdominal pain and vomiting for 11 days. The patient experienced mild-to-moderate abdominal pain without fever. Blood tests revealed a white blood cell count of 2.7*10^9/L within the normal range, with low hemoglobin and platelet levels. The monocyte percentage was 10.4%, absolute neutrophil count was 1.2*10^9/L, and C-reactive protein level was 121.9 mg/L. The levels of total bile acids (10.5 μmol/L), aspartate aminotransferase (71 U/L), alanine aminotransferase (56 U/L), and alkaline phosphatase (178 U/L) were elevated. Serum carcinoembryonic antigen (5.8 ng/mL), cytokeratin 19 fragment (13.8 ng/mL), and carbohydrate antigen 12–5 (12.5 ng/mL) were elevated, while carbohydrate antigen 19–9 remained within normal limits (see Table 2). The patient had been diagnosed with a pulmonary malignancy 2 years prior and had been regularly taking anlotinib (1 tablet daily). The patient denied a history of internal diseases such as hypertension, diabetes, heart disease, or kidney disease. Contrast-enhanced abdominal computed tomography (CT) scan showed significant gallbladder dilation (18.6 × 7.2 × 5.3 cm) with slight thickening and irregularity of the gallbladder wall. Additionally, magnetic resonance cholangiopancreatography and contrast-enhanced magnetic resonance imaging revealed mild dilation of the hepatic bile duct and cystic duct, while ruling out biliary stone impaction or tumor-like lesions in the biliary system. The final diagnosis was acute cholecystitis complicated with GGB (Figure 1).

**Table 2:** Laboratory investigations upon hospitalization.

Blood markers	Course of treatment			Normal range
	Index^a^	Index^b^	Index^c^	
Leucocytes (×10^9^/L)	2.7	3.7	4.0	3.5–9.5
Hemoglobin (g/L)	105	107	117	115–150
Platelets (×10^9^/L)	110	127	137	125–350
CRP(mg/L)	121.9	27.7	5.0	0–8
ALT (U/L)	13	14	11	0–40
AST (U/L)	71	31	33	0–35
GGT (U/L)	56	44	15	0–45
ALP(U/L)	178	75	90	35–100
TBIL (μmol/L)	9.5	6.6	7.7	3.42–20.5
DBIL (μmol/L)	4.8	2.0	1.4	0–6.42
TBA (μmol/L)	10.5	7.8	5.0	0–10
GLU (mmol/L)	4.8	3.9	4.1	3.9–6.1
Urea (μmol/L)	4.6	1.9	4.5	1.7–7.5
Cr (μmol/L)	72	66	61	44–120
PA (mg/L)	175	186	188	170–420
PT (sec)	9.7	10.1	11.1	9–13
APTT (sec)	25.6	27.1	26.3	25–37
CA125	37.9	NR	NR	0–35
CA19-9	1.2	NR	NR	0–37
AFP	1.0	NR	NR	0–8
CEA	5.8	NR	NR	0–5
CYFRA21-1	13.8	NR	NR	0–2.08

a Initial inspection.

b Three days after antibiotic treatment.

c Seven days after antibiotic treatment.

ALT, alaniene transaminase; AST, aspartate transaminase; ALP, alkaline phosphatase; GGT, g-glutamyl transferase; TBIL, total bilirubin; DBIL, direct bilirubin; TBA, total bile acids; GLU, glucose; Cr, creatinine; PA, prealbumin; PT, prothrombin time; APTT, activated partial thromboplastin time; NR, not record.

**Figure 2:**
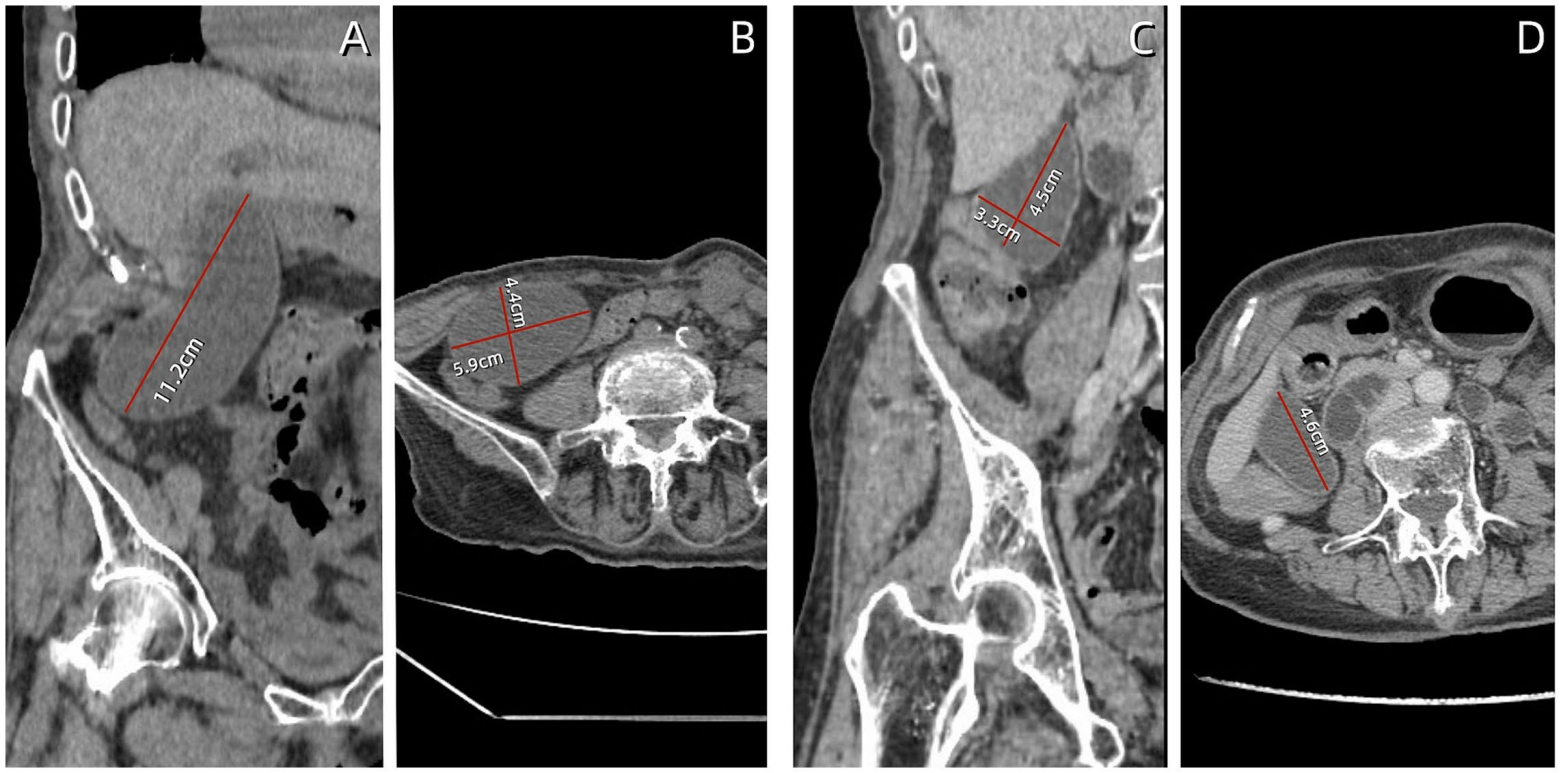
Follow-up abdominal computed tomography (CT) findings. **(A,B)** After 3 days of antibiotic treatment, the gallbladder volume decreased, measuring approximately 11.2 × 5.9 × 4.4 cm. **(C,D)** After 7 days of antibiotic treatment, the gallbladder returned to normal size, measuring approximately 4.6 × 4.5 × 3.3 cm.

Given the patient’s impaired liver function and abnormally elevated inflammatory marker levels, anlotinib was discontinued and antibiotic therapy was initiated. Three days after intravenous administration of cefoperazone sodium and sulbactam (2 g/q12 h), the patient’s vomiting symptoms resolved, abdominal pain was alleviated, and blood tests revealed a C-reactive protein (CRP) level of 27.7 mg/L. Total bile acid, aspartate aminotransferase, alanine aminotransferase, and alkaline phosphatase levels decreased to within normal ranges. Abdominal CT revealed a significant reduction in gallbladder size (11.2 × 5.9 × 4.4 cm), with no dilation observed in the intrahepatic bile ducts or cystic duct (Figures 2A,B). Following 1 week of antibiotic treatment, the patient’s abdominal pain symptoms nearly disappeared, and CRP levels decreased to the normal range (5.0 mg/L). CT confirmed that the gallbladder had returned to its normal size (Figures 2C,D). The patient was discharged after 3 days. Follow-up examination 2 weeks later in the general surgery clinic showed good recovery, and abdominal ultrasound examination did not show recurrence of gallbladder swelling. The strategy of trying to replace new anlotinib was adopted.

## Discussion

Generally, the gallbladder capacity of a normal adult does not exceed 60 mL. In clinical practice, it is relatively common for various pathological conditions to cause gallbladder enlargement of 250–350 mL (4). However, reports on extreme gallbladder sizes are rare. Due to the lack of clear clinical or histological definitions, some scholars define a gallbladder with a diameter >14 cm and a capacity ≥1.5 L as GGB (5). The pathogenesis of GGB remains unclear. We reviewed multiple literature sources that conducted in-depth research on the occurrence mechanisms of GGB. The Taxonera biliary valve mechanism is the mainstream theory explaining mechanical obstruction-induced GGB. Chronic biliary obstruction caused by stones or tumors, particularly in patients with progressive diseases, such as malignant tumors, leads to long-term elevated intraductal pressure, resulting in gallbladder enlargement (6). Some scholars argue that, while gallstones can cause intermittent obstruction, they are insufficient to chronically sustain elevated intraductal pressure. Moreover, long-standing gallstones may lead to gallbladder atrophy and fibrosis, which limits enlargement (7, 8). Beyond obstructive factors, Kuznetsov proposed that other non-obstructive factors may alter the gallbladder drainage capacity, such as neurological, hydraulic, or combined congenital organ dysfunction. These cases often involve younger patients (4). For instance, Maeda reported a case of a 36-year-old GGB patient primarily presenting with progressive gallbladder enlargement without clinical complications, ultimately diagnosed with focal ganglion cell deficiency in the gallbladder neck (9). In addition to congenital gallbladder abnormalities, patients with type II diabetes exhibit significantly reduced gallbladder wall receptor sensitivity to cholecystokinin due to obesity, coupled with excessive somatostatin secretion. This dual mechanism suppresses normal production of cholecystokinin and cholecystokinin-like substances (cholagogues), thereby impairing gallbladder contraction (10, 11). Furthermore, some circumstances may be paraphysiological such as elevated progesterone levels during pregnancy or hormone replacement therapy which can damage gallbladder contractility. These conditions delay bile emptying, alter bile composition, and promote the precipitation of cholesterol and bilirubin calcium crystals, which facilitate gallstone formation. Chronic irritation from these stones may ultimately cause gallbladder enlargement and even acute pancreatitis (12, 13). A case of GGB associated with a genetic mutation was reported by Panaro et al. in 2012, which involved a 17-year-old adolescent preparing for liver transplantation because of chronic liver disease. Genetic testing revealed a mutation in the ABCB11 gene, confirming the diagnosis of progressive familial intrahepatic cholestasis type 2 (14). Mutations in the ABCB11 gene impair the activity of the bile salt export pump, resulting in bile retention in the biliary tract. This is the largest reported case of GGB, with the gallbladder reaching an astonishing size of 43 × 21 × 20 cm. Unlike the etiological analysis of the GGB cases reported above, we reported the first case of GGB caused by adverse reactions to chemotherapy drugs.

The patient in this report is an elderly female with malignant lung tumor who had been taking anlotinib orally for 2 years on a regular basis, with no history of internal diseases such as hypertension, diabetes, heart disease, or kidney disease. Laboratory tests revealed impaired liver function and abnormally elevated levels of blood inflammatory markers. Imaging examinations revealed acute cholecystitis with GGB, clearly ruling out biliary tract stones and space-occupying lesions causing biliary obstruction. After discontinuing the chemotherapy drug and administering antibiotic treatment, the gallbladder returned to its normal size, and the patient’s liver function and inflammatory markers normalized. The Naranjo scale was used to assess whether the patient’s acute cholecystitis with GGB was caused by anlotinib. This scale is primarily used to evaluate the likelihood of drug-related adverse events (15). Our patient scored five points, indicating that the occurrence of acute cholecystitis with GGB was likely associated with the use of anlotinib (Table 3). Anlotinib is a multi-target tyrosine kinase inhibitor (TKI) primarily used to treat non-small cell lung cancer. It exerts anti-tumor effects by inhibiting the expression of vascular endothelial growth factor, fibroblast growth factor, and stem cell factor receptors, thereby suppressing tumor angiogenesis and tumor cell proliferation (16). TKI directly inhibits the activity of activated enzymes and changes the expression spectrum of miRNA in cells. MiRNA is the core regulator of angiogenesis, and some miRNA participate in angiogenesis by directly targeting proangiogenic factors or receptors (17). For example, miR-29 inhibits angiogenesis by downregulating VEGF overexpression, while miR − 195 promotes angiogenesis and metastasis by enhancing the expression of VEGF and its metastasis-promoting factors. Beyond directly targeting VEGF, certain miRNAs guide mature endothelial cells to migrate into hypoxic environments by inhibiting VEGF-inducible factors (such as miR-22, miR − 107, miR-519c, and miR − 145 in the HIF-1 pathway). This ultimately achieves tumor growth inhibition (18–21). Inevitably, while inhibiting tumor angiogenesis, anlotinib also suppresses blood vessel growth in healthy organs such as the gallbladder (22). Although there is currently no definitive evidence or widespread reports indicating that anlotinib causes inflammatory changes in the gallbladder, there are numerous reports of cholecystitis or gallbladder abnormalities associated with other TKI, such as sorafenib and sunitinib (3, 23). The pharmacological mechanisms underlying TKI-induced gallbladder toxicity are complex and not yet fully elucidated; however, they are generally considered to result from multifactorial effects, primarily involving the following aspects: 1. Target inhibition: One of the main targets of many TKIs (e.g., sunitinib and sorafenib) is the vascular endothelial growth factor receptor. Vascular endothelial growth factor receptor inhibition leads to impaired vascular endothelial cell function, affecting blood supply to the gallbladder wall (microcirculatory disturbance). Gallbladder wall ischemia compromises gallbladder smooth muscle function, resulting in weakened contractility and delayed emptying. 2. Alterations in the bile composition: TKIs may interfere with bile production and secretion by inhibiting bile acid transporter enzymes in the hepatocytes. The abnormal elevation in bile acid concentration can exert direct chemical irritation and toxic effects on the gallbladder mucosa, thereby inducing inflammation. 3. Direct cytotoxic effects: Some TKIs or their metabolites may exert direct toxic effects on the gallbladder mucosal epithelial cells, compromising the integrity of the mucosal barrier. Cellular damage releases inflammatory mediators, triggering local inflammatory responses that attract inflammatory cell infiltration, resulting in gallbladder wall edema, thickening, and inflammation. 4. Secondary effects of liver injury: TKI-induced drug-related liver injury directly affects bile production and excretion, leading to intrahepatic cholestasis. This increases the viscosity of bile entering the gallbladder and may cause reflux, which affects gallbladder function, indirectly promoting cholecystitis and gallbladder enlargement (16, 24, 25).

**Table 3:** Adverse drug reaction probability scale (Naranjo scale) in the present case.

Question	Yes	No	Do not know	Score
1. Are there previous conclusive reports on this reaction?	1	0	0	0
2. Did the adverse event appear after the suspected drug was given	2	−1	0	2
3. Did the adverse reaction improve when the drug was discontinued or a specific antagonist was given?	1	0	0	1
4. Did the adverse reaction appear when the drug was re-administered?	2	−1	0	0
5. Are there alternative causes that could have caused the reaction?	−1	2	0	2
6. Did the reaction reappear when a placebo was given?	−1	1	0	0
7. Was the drug detected in any body fluid in toxic concentrations?	1	0	0	0
8. Was the reaction more severe when the dose was increased, or less severe when the dose was decreased?	1	0	0	0
9. Did the patient have a similar reaction to the same or similar drugs in any previous exposure?	1	0	0	0
10. Was the adverse event confirmed by any objective evidence?	1	0	0	0
Scoring				5

Currently, there are no management guidelines for giant gallbladder (GGB) both domestically and internationally. The clinical management principles for GGB generally involve first identifying the underlying causes of extreme gallbladder enlargement, followed by standardized treatment according to corresponding etiology guidelines. Regarding etiology, we have reviewed and summarized the latest expert consensus and management guidelines from domestic and international sources, supplemented by Table 4. Laparoscopic cholecystectomy (LC) is considered the preferred surgical option for benign GGB cases with identifiable etiologies. Notably, antibiotic therapy serves as an effective supportive treatment for first-time GGB patients and helps control postoperative infections.

**Table 4:** Etiological management guidelines for GGB.

Etiology	Management	Follow up	Reference
Benign cause			
Biliary obstruction (e.g., impacted stones)	Obstruction relief: medication for stone expulsion in mild cases; cholecystectomy with primary LC for severe or impacted cases	US/CT review at 1 month and 6 months after surgery	(29–32)
Acute cholecystitis	For mild cases, cholecystectomy should be performed as early as possible within 1 week. First, LC is the best time within 72 h. Intermediate and severe cases: timely anti-infection and systemic supportive treatment, cholecystectomy or cholecyst drainage within 24 h (PTGBD is preferred).	US/CT review at 1 month and 3 months after surgery	(29–32)
Chronic cholecystitis	Anti-inflammatory and choleretic; if recurrent or gallbladder function is lost, cholecystectomy (first LC).	Nonoperative treatment: 6–12 months US review. Surgical treatment: US/CT review at 1 month and 3 months after surgery.	(29–32)
Congenital gallbladder abnormalities (e.g., congenital biliary cyst, congenital gallbladder morphological variation)	In cholecystectomy, biliary tract structure changes occur first and then biliary reconstruction (such as Roux-en-Y anastomosis) is performed.	Long-term follow-up, yearly.	(33–35)
Malignant cause			
Carcinoma of gallbladder	Radical surgical resection (including gallbladder, partial liver tissue and lymph node clearance), postoperative adjuvant chemotherapy.	CT and tumor markers were reviewed every 3 months after surgery.	(36, 37)

GGB, giant gallbladder; LC, laparoscopic cholecystectomy; PTGBD, percutaneous transhepatic gallbladder drainage; US, ultrasound; CT, computer tomography.

This case report has certain limitations. First, due to the patient’s poor physical condition, they refused cholecystectomy, preventing us from obtaining histological results for in-depth exploration of the specific pathological mechanisms of anlotinib-induced gallbladder toxicity. We will conduct extended research through long-term follow-up observations and collect similar cases from other hospitals or regions for comprehensive analysis. Second, the case did not undergo multidisciplinary consultation to formulate the optimal treatment plan. In future, we will integrate resources from oncology, hepatobiliary surgery, gastroenterology, and radiology departments to establish a personalized multidisciplinary diagnosis and treatment system. Meanwhile, we will advance basic and translational medical research through animal models or organoid technology to precisely decipher the molecular mechanisms of anlotinib-induced gallbladder injury, identify key signaling pathways, and provide crucial evidence for targeted preventive drug development.

In summary, during the treatment of malignant tumors with anlotinib, when patients present with persistent gallbladder enlargement accompanied by cholecystitis, it is necessary to first clarify the true etiology of extreme gallbladder enlargement. Once biliary obstruction is ruled out as a causative factor of GGB, anlotinib can be discontinued while administering antibiotic therapy. After the gallbladder size and inflammatory marker levels return to the normal ranges, anlotinib treatment can be resumed. The entire process typically requires only a few days for completion.

## Conclusion

To our knowledge, this is the first reported case of GGB associated with adverse reactions to anlotinib. By sharing the diagnosis and treatment experience of this case, we hope to provide new insights into the etiology of GGB and to alert clinicians to remain vigilant against such adverse events when using TKI-class chemotherapy drugs.

## Data Availability

The raw data supporting the conclusions of this article will be made available by the authors, without undue reservation.

## References

[1] Gao Y He D Feng W Yue J Jian Z. Laparoscopic cholecystectomy for giant gallbladder: a case report. Medicine. (2023) 102:e35429. doi: 10.1097/MD.0000000000035429, PMID: 37800759 PMC10553151

[2] Wang L He Z Yang S Tang H Wu Y Li S. The impact of previous therapy strategy on the efficiency of anlotinib hydrochloride as a third-line treatment on patients with advanced non-small cell lung cancer (NSCLC): a subgroup analysis of ALTER0303 trial. Transl Lung Cancer Res. (2019) 8:575–83. doi: 10.21037/tlcr.2019.09.21, PMID: 31737494 PMC6835105

[3] Zhong Q Liu Z. Efficacy and safety of anlotinib in patients with advanced non-small cell lung cancer: a real-world study. Cancer Manag Res. (2021) 13:4115–28. doi: 10.2147/CMAR.S304838, PMID: 34045898 PMC8149213

[4] Kuznetsov AV Borodach AV Fedin EN Khromova AD. Giant gallbladder: a case report and review of literature. Int J Surg Case Rep. (2014) 5:673–6. doi: 10.1016/j.ijscr.2014.08.005, PMID: 25194602 PMC4189069

[5] Zong L Chen P Wang L He C Wang G Jiang J. A case of congenital giant gallbladder with massive hydrops mimicking celiac cyst. Oncol Lett. (2013) 5:226–8. doi: 10.3892/ol.2012.1010, PMID: 23255925 PMC3525506

[6] Taxonera Samso C García Albarrán J Villacorta Patinoˇ J Diaz-Rubia GM. Vesícula gigante: Aportatión de un caso y revisión de la literatura. Rev Esp Enferm Apar Dig. (1983) 64:141–4. PMID: 6635300

[7] Singal R Gupta S Singal RP Mittal A Sharma S Khurana R. Gall bladder perforation leads to liver abscess formation – role of ultrasonography. J Gastrointest Dig Syst. (2015) 5:279. doi: 10.4172/2161-069X.1000279, PMID: 39887974

[8] Woywodt A Matteson E. Should eponyms be abandoned? Yes BMJ. (2007) 335:424. doi: 10.1136/bmj.39308.342639.AD, PMID: 17762033 PMC1962844

[9] Maeda Y Setoguchi T Yoshida T Katsuki T. A giant gallbladder. Gastroenterol Jpn. (1979) 14:621–4. doi: 10.1007/BF02773722, PMID: 527798

[10] Fitzgerald JE White MJ Lobo DN. Courvoisier’s gallbladder: law or sign? World J Surg. (2009) 33:886–91. doi: 10.1007/s00268-008-9908-y, PMID: 19190960

[11] Calomino N Poto GE Carbone L Bagnacci G Piccioni S Andreucci E. Neuroendocrine tumors' patients treated with somatostatin analogue could complicate with emergency cholecystectomy. Ann Ital Chir. (2023) 94:518–22. Available at: https://pubmed.ncbi.nlm.nih.gov/38051513/ PMID: 38051513

[12] Uemura S Namikawa T Uchida K Hanazaki K. Gastrointestinal: Giant gallbladder. J Gastroenterol Hepatol. (2022) 37:2206. doi: 10.1111/jgh.15858, PMID: 35535652

[13] Veres M Flamind Oltean S Pascanu S Butiulca M Branea OE Lazar AE. Too late to reverse: an atypical postpartum case of acute necrotizing pancreatitis with refractory ARDS despite ECMO support. Life. (2025) 15:1347. doi: 10.3390/life15091347 41010289 PMC12471077

[14] Panaro F Chastaing L Navarro F. Hepatobiliary and pancreatic: Giant gallbladder associated with Byler’s disease. J Gastroenterol Hepatol. (2012) 27:620. doi: 10.1111/j.1440-1746.2012.07058.x, PMID: 22353353

[15] Naranjo CA Busto U Sellers EM Sandor P Ruiz I Roberts EA. A method for estimating the probability of adverse drug reactions. Clin Pharmacol Ther. (1981) 30:239–45. doi: 10.1038/clpt.1981.154, PMID: 7249508

[16] Aihara Y Yoshiji H Yamazaki M Ikenaka Y Noguchi R Morioka C. A case of severe acalculous cholecystitis associated with sorafenib treatment for advanced hepatocellular carcinoma. World J Gastrointest Oncol. (2012) 4:115–8. doi: 10.4251/wjgo.v4.i5.115, PMID: 22645635 PMC3360105

[17] Giuppi M La Salvia A Evangelista J Ghidini M. The role and expression of angiogenesis-related miRNAs in gastric Cancer. Biology (Basel). (2021) 10:146. doi: 10.3390/biology10020146, PMID: 33673057 PMC7918665

[18] Yamakuchi M Yagi S Ito T Lowenstein CJ. MicroRNA-22 regulates hypoxia signaling in colon cancer cells. PLoS One. (2011) 6:e20291. doi: 10.1371/journal.pone.0020291, PMID: 21629773 PMC3100326

[19] Yamakuchi M Lotterman CD Bao C Hruban RH Karim B Mendell JT. P53-induced microRNA-107 inhibits HIF-1 and tumor angiogenesis. Proc Natl Acad Sci USA. (2010) 107:6334–9. doi: 10.1073/pnas.0911082107, PMID: 20308559 PMC2851979

[20] Cha ST Chen PS Johansson G Chu CY Wang MY Jeng YM. MicroRNA-519c suppresses hypoxia-inducible factor-1alpha expression and tumor angiogenesis [published correction appears in cancer res. 2019; 79 (14): 3790]. Cancer Res. (2010) 70:2675–85. doi: 10.1158/0008-5472.CAN-09-2448, PMID: 20233879

[21] Zhang H Pu J Qi T Qi M Yang C Li S. MicroRNA-145 inhibits the growth, invasion, metastasis and angiogenesis of neuroblastoma cells through targeting hypoxia-inducible factor 2 alpha. Oncogene. (2014) 33:387–97. doi: 10.1038/onc.2012.574, PMID: 23222716

[22] Sanda M Tamai H Deguchi H Mori Y Moribata K Shingaki N. Acalculous cholecystitis in a patient with hepatocellular carcinoma on sorafenib. ISRN Gastroenterol. (2011) 2011:201529. doi: 10.5402/2011/201529, PMID: 21991498 PMC3168393

[23] Choi SW Lee JM Kim DG Noh MH. Acute acalculous cholecystitis associated with sunitinib treatment for renal cell carcinoma. Korean J Gastroenterol. (2020) 75:103–7. doi: 10.4166/kjg.2020.75.2.103 32098465

[24] Di Stefano M Colombo C De Leo S Perrino M Viganò M Persani L. High prevalence and conservative management of acute cholecystitis during Lenvatinib for advanced thyroid cancer. Eur Thyroid J. (2021) 10:314–22. doi: 10.1159/000510369, PMID: 34395303 PMC8314780

[25] Tirumani SH Krajewski KM Shinagare AB Jagannathan JP Ramaiya NH. Gallbladder complications associated with molecular targeted therapies: clinical and imaging features. Clin Imaging. (2014) 38:50–5. doi: 10.1016/j.clinimag.2013.08.012, PMID: 24135148

[26] Borodach AV Borodach VA Kim AN. Gigantskaya vodyanka zhelchnogo puzyrya. Novosibirsk: Sibirskiy Universitet; (2005). p. 62–63.

[27] Fultang J Chinaka U Ali A. Giant gallbladder presenting as a right iliac Fossa mass removed by Mini-laparoscopic cholecystectomy. Cureus. (2019) 11:e5576. doi: 10.7759/cureus.5576, PMID: 31695995 PMC6820684

[28] Mirali H Kamaoui I El Harroudi T Skiker I Serji B. Giant gallbladder revealed by chronic cholecystitis gallstone: a case report and review of the literature. Cureus. (2021) 13:e13906. doi: 10.7759/cureus.13906, PMID: 33880262 PMC8046698

[29] de’Angelis N Catena F Memeo R Coccolini F Martínez-Pérez A Romeo OM. 2020 WSES guidelines for the detection and management of bile duct injury during cholecystectomy. World J Emerg Surg. (2021) 16:30. doi: 10.1186/s13017-021-00369-w, PMID: 34112197 PMC8190978

[30] Okamoto K Suzuki K Takada T Strasberg SM Asbun HJ Endo I. Tokyo guidelines 2018: flowchart for the management of acute cholecystitis [published correction appears in J hepatobiliary Pancreat Sci. 2019; 26 (11): 534. Doi:10.1002/jhbp.686.]. J Hepatobiliary Pancreat Sci. (2018) 25:55–72. doi: 10.1002/jhbp.516, PMID: 29045062

[31] Vera K Pei KY Schuster KM Davis KA. Validation of a new American Association for the Surgery of Trauma (AAST) anatomic severity grading system for acute cholecystitis. J Trauma Acute Care Surg. (2018) 84:650–4. doi: 10.1097/TA.0000000000001762, PMID: 29271871

[32] Ansaloni L Pisano M Coccolini F Peitzmann B Fingerhut A Catena F. WSES guidelines on acute calculous cholecystitis [published correction appears in world]. World J Emerg Surg. (2016) 11:25. doi: 10.1186/s13017-016-0082-5 27307785 PMC4908702

[33] Shi J Li K Chen Y. Rare giant bile duct cyst with new-onset liver neoplasm nine years after surgery: a cast report. Chin J Hepatic Surg. (2025) 14:126–7. doi: 10.3877/cma.j.issn.2095-3232.2025001

[34] Keplinger KM Bloomston M. Anatomy and embryology of the biliary tract. Surg Clin North Am. (2014) 94:203–17. doi: 10.1016/j.suc.2014.01.001, PMID: 24679417

[35] Paraf F Lemaigre G Bedossa P. Vésicule biliaire trilobée [Trilobed gallbladder]. Gastroenterol Clin Biol. (1987) 11:713–4. PMID: 3692098

[36] Vogel A Bridgewater J Edeline J Kelley RK Klümpen HJ Malka D. Biliary tract cancer: ESMO clinical practice guideline for diagnosis, treatment and follow-up. Ann Oncol. (2023) 34:127–40. doi: 10.1016/j.annonc.2022.10.506, PMID: 36372281

[37] Benson AB D'Angelica MI Abrams T Abbott DE Ahmed A Anaya DA. NCCN guidelines® insights: biliary tract cancers, version 2.2023. J Natl Compr Cancer Netw. (2023) 21:694–704. doi: 10.6004/jnccn.2023.0035, PMID: 37433432

